# Modern Homœopathy

**Published:** 1869-08

**Authors:** 


### Modern Homœopathy.

The *Medical and Surgical Reporter* (Phila.,) furnishes the following extract from the proceedings of the Cleveland Homœopathic Medical Society, recently held in that city:

"Dr. S. R. Beckwith asked if the members had any experience to report on the use of bromide of potassium in epilepsy; said it was a pretty sure remedy, given in *sensible* doses. He related several cases favorably affected by its use.

"Dr. P. Wilson reported that late clinical reports had shown that in bad cases of epilepsy, it was safe to give as high as *sixty grains of bromide of potassium three times daily;* that such doses caused temporary insanity, which might be continued many weeks, and yet disappear on ceasing the use of the medicine."

The same Journal says, that the reports of the London Homœopathic hospitals show a decided partiality for similar "massive" doses.—*Medical and Surgical Journal.*

